# Cognitive, Behavioral, and Sensory Profile of Pallister–Killian Syndrome: A Prospective Study of 22 Individuals

**DOI:** 10.3390/genes13020356

**Published:** 2022-02-16

**Authors:** Anna Fetta, Luca Soliani, Alessia Trevisan, Rosa Pugliano, Emilia Ricci, Veronica Di Pisa, Veronica Pignataro, Marida Angotti, Alessandro Rocca, Bianca Salce, Maria Margherita Mancardi, Lucio Giordano, Dario Pruna, Antonia Parmeggiani, Duccio Maria Cordelli

**Affiliations:** 1 IRCCS Istituto delle Scienze Neurologiche di Bologna, UOC di Neuropsichiatria dell’Età Pediatrica, 40139 Bologna, Italy; anna.fetta@studio.unibo.it (A.F.); luca.soliani2@unibo.it (L.S.); rosa.pugliano@studio.unibo.it (R.P.); veronica.dipisa@aosp.bo.it (V.D.P.); veronica.pignataro@libero.it (V.P.); marida.angotti@hotmail.it (M.A.); bianca.salce@studio.unibo.it (B.S.); antonia.parmeggiani@unibo.it (A.P.); 2 Dipartimento di Scienze Mediche e Chirurgiche (DIMEC), Università di Bologna, 40138 Bologna, Italy; alessia.trevisan@studio.unibo.it; 3 Child Neuropsychiatry Unit, Epilepsy Center, Ospedale San Paolo, Dipartimento di Scienze della Salute, Università di Milano, 98051 Milan, Italy; emiliaricci85@gmail.com; 4 UO di Pediatria d’Urgenza, IRCCS Policlinico Sant’Orsola, 40138 Bologna, Italy; alessandro.rocca4@unibo.it; 5 Child Neuropsychiatry Unit, Department of Medical and Surgical Neurosciences and Rehabilitation, IRCCS Istituto Giannina Gaslini, 16128 Genova, Italy; margheritamancardi@ospedale-gaslini.ge.it; 6 Child Neuropsychiatric Division, Spedali Civili, 25123 Brescia, Italy; lucio.giordano@asst-spedalicivili.it; 7 Department of Pediatric Neurology and Epileptology, Pediatric Depatment, ARNAS Brotzu, 09134 Cagliari, Italy; dario.pruna@aob.it

**Keywords:** PKS, tetrasomy 12p, Sensory Profile 2, Bayley-3, Vineland-II, stereotypies

## Abstract

Background: Developmental delay and intellectual disability are two pivotal elements of the phenotype of Pallister–Killian Syndrome (PKS). Our study aims to define the cognitive, adaptive, behavioral, and sensory profile of these patients and to evaluate possible correlations between the different aspects investigated and with the main clinical and demographic variables. Methods: Individuals of any age with genetically confirmed PKS were recruited. Those ≤ 42 months were administered the Bayley Scales of Infant and Toddler Development Third Edition (Bayley-III), and those > 42 months the Vineland Adaptive Behavior Scales—Second Edition (Vineland-II). Stereotyped behaviors (Stereotypy Severity Scale, SSS) and aggressive behaviors (Behavior Problems Inventory—Short Version, BPIs) were assessed in all subjects > 1 year; sensory profile (Child Sensory Profile 2, C-SP2) in all aged 2–18 years. Results: Twenty-two subjects were enrolled (11 F/11 M; age 9 months to 28 years). All subjects ≤ 42 months had psychomotor developmental delay. Of the subjects > 42 months, 15 had low IQ deviation, and 1 in the normal range. Stereotypies were frequent (median SSS-total score 25/68). Lower Vineland-II values corresponded to greater intensity and frequency of stereotypies (*p* = 0.004 and *p* = 0.003), and self-injurious behaviors (*p* = 0.002 and *p* = 0.002). Patients with severe low vision had greater interference of stereotypies (*p* = 0.027), and frequency and severity of aggressive behaviors (*p* = 0.026; *p* = 0.032). The C-SP2, while not homogeneous across subjects, showed prevalence of low registration and sensory seeking profiles and hypersensitivity to tactile and auditory stimuli. Lower Vineland-II scores correlated with higher Registration scores (*p* = 0.041), while stereotypies were more frequent and severe in case of high auditory sensitivity (*p* = 0.019; *p* = 0.007). Finally, greater sleep impairment correlated with stereotypies and self-injurious behaviors, and lower Vineland-II scores. Conclusions: The present study provides a further step in the investigation of the etiopathogenesis of the syndrome. Furthermore, these aspects could guide rehabilitation therapy through the identification of targeted protocols.

## 1. Introduction

Pallister–Killian Syndrome (PKS) is a rare genetic disorder with multisystemic involvement caused by the tissue-limited mosaicism of 12p supernumerary isochromosome [1,2,3,4]. Neurologic phenotype includes epilepsy [5,6], sleep disturbances, cerebral malformation [7], neuropsychological delay, and intellectual disability [8]. Although most of the diagnosed individuals have profound intellectual disabilities and severe developmental delay [3,8], individuals with mild impairment have been described [9,10,11,12], suggesting the existence of a wider spectrum, less known because of underdiagnosis of subtle phenotypes.

To date, only one study in literature has explored behavioral and cognitive features on a cohort of 14 PKS patients aged 16 months to 19 years, using, among others, the Vineland Adaptive Behavior Scales—Second Edition (Vineland-II), the Aberrant Behavior Checklist, the Modified Checklist for Autism in Toddlers, and the Social Communication Questionnaire [8]. Most of the patients in that cohort had intellectual disability and/or developmental delay. Repetitive and self-injurious behavior were detected, as well as lethargy and withdrawal, tactile defensiveness and hypotonia. Autistic features were suspected, although data proved inconclusive in consideration of the severity of the intellectual disability of the children examined. Moreover, various degrees of hearing and visual impairment were present in most of the population [8]. No assessment of stereotypies nor of the neurosensory profile was performed. No correlation between the different aspects examined was evaluated.

Sensory processing is the integration of information from peripheral sensory receptors (exteroception and proprioception) by the central nervous system (CNS) in order to modulate the adaptive response to the demands of everyday life [13]. Different individuals, even those with normotypic development, have different sensory profiles [14]. Alterations in sensory processing are known to be present in autism spectrum disorder (ASD) [15,16,17,18]. More recently they have been related to attention deficit hyperactive disorder (ADHD) [19] and intellectual developmental disability [20]. Interestingly, also genetic syndromes such as Down syndrome [21,22], Angelman syndrome, and Cornelia de Lange syndrome [23] have characteristic altered sensory profile (ASP), as well as individuals with corpus callosum malformation [24].

Moreover, repetitive behaviors (RB) are frequent in syndromes such as Down syndrome, Prader–Willi syndrome, and William syndrome [25], and have been described in PKS by Kostanecka [8].

ASP are known to be strictly related to RB both in idiopathic ASD and in genetic syndromes [15,26].

Our study aims to systematically investigate cognitive, adaptive, behavioral, and sensory profile in PKS syndrome and to identify any possible correlation between these aspects and with the main clinical and demographic features.

## 2. Materials and Methods

### 2.1. Study Design

For this prospective study, individuals of any age with a genetically confirmed diagnosis of PKS were prospectively enrolled in collaboration with the Association PKS Kids Italy ONLUS.

Neuropsychological assessment was performed by experienced neuropsychologists or clinicians either in person or through telemedicine as the circumstances dictated.

Test results, as well as clinical and demographic data were collected in an anonymized database and examined.

### 2.2. Neuropsychological Assessment

#### 2.2.1. Developmental and Adaptive Profile

To evaluate the cognitive and adaptive profile in subjects ≤ 42 months, The Bayley Scales of Infant and Toddler Development Third Edition (Bayley-III) was administered. For those aged 42 months to 18 years, Vineland-II was administered in consideration of the greater reliability of adaptive tests in older individuals with severe intellectual disabilities [8].

The Bayley-III is an ability test of global development. Cognitive, language, and motor domains are assessed through behavioral assessment while social-emotional and adaptive behaviors are evaluated through parent report. Moreover, it comprises a motor score, and fine and gross motor subtests.

The Vineland-II is a semi-structured interview for parents or caregivers that provides an overall measure of adaptive behavior ability. It consists of 4 scales, subdivided into 11 subscales; for each, raw scores are converted according to the age, and a deviance IQ score is obtained (mean 100, SD 15; range 20–160). The Adaptive Behavior Composite (ABC) score is obtained from the sum of the IQ scores of the subscales and converted to deviance IQ. Scores are then labeled as “low” (20–70), “moderately low” (71–85), “adequate” (86–114), “moderately high” (115–129), and “high” (130–140).

#### 2.2.2. Behavioral Profile

Stereotyped behaviors were assessed in all subjects aged > 1 year using the stereotypy severity scale (SSS) and the behavior problems inventory-short form (BPIs).

The SSS investigates the purely motor stereotypies component and considers 4 dimensions: number (0–3 points), frequency (0–5 points), intensity (0–5 points), and interference (0–5 points). An independent rating of global impairment caused by the movement (0–50) is added to obtain the total score (up to 68 points, indicating the worst severity and impairment) [27].

For aggressive behaviors, we used 2 of the 3 subscales of the BPIs (a shortened version of the BPI-01): Self-Injurious Behavior and Aggressive/Destructive Behavior, analyzing for each the frequency and intensity [28,29].

#### 2.2.3. Sensory Profile

Sensory profile was assessed in all subjects aged 2–18 years using the Child Sensory Profile 2 (C-SP2). The questionnaire is composed of 86 items, corresponding to principal domains of sensory processing, modulation, behavior in response to stimuli, and response style which is obtained by summing items belonging to different sections. The Likert scoring of the C-SP2 represents “0 = Not Applicable and then 1 = Almost Never” to “5 = Almost Always”.

In Dunn’s sensory processing framework, thresholds range from high (slow to detect) to low (quick to detect), and self-regulation ranges from passive to active. Four sensory processing patterns are then identified: Registration (also referred to as hypo-responsiveness, characterized by high threshold and passive self-regulation), Seeking (high threshold and active self-regulation), Sensitivity (low threshold and passive self-regulation), and Avoiding (low threshold and active self-regulation). Moreover, it recognizes six sensory modalities (i.e., Auditory, Visual, Touch, Movement, Body Position and Oral), and three behavioral sections (i.e., Conduct, Attention and Social). The SP-2 classifies children as having “typical performance” “less”/”more” than others (between 1 and 2 SD) or a “much less”/”much more” than others (>2 SD) [14,30].

### 2.3. Characterization of Repetitive Behaviors through Direct Video Analysis

Videos performed during EEG prolonged monitoring, video polysomnographic (VPSG), or awake video-EEG recording as well as home videos recordings were collected. Video analysis was performed using a coding sheet developed both on the basis of Jankovic’s definition of stereotypy [31] and the categories described in other syndromes with characteristic movement disorders and especially stereotypies [32]. We recorded the type of movement seen and the body part involved.

### 2.4. Sleep

Data about sleep impairment measured through the Sleep Disturbance Scale for Children (SDSC) and previously used for the study of sleep in PKS were also collected [33]. Patients who had not participated in that study were administered the test.

### 2.5. Ethics

Each person involved received and completed the forms dedicated to informed consent, participation, study, and treatment of personal data.

### 2.6. Data Analysis

In the descriptive analysis, continuous variables were presented through median and interquartile range (IQR), categorical variables through absolute numbers and percentages.

The relationships between the scores of the different tests were evaluated with the Spearman Rho coefficient.

The comparison of the different scores according to demographic and clinical variables was performed with the Mann–Whitney’s U test. To define the possible influence of age in the behavioral and sensory profile, two groups were identified on the basis of the median age of the group of patients tested for it.

The classic statistical significance threshold *p* < 0.05 was set.

## 3. Results

### 3.1. Population

Twenty-two subjects (11 females/11 males; age range 9 months–28 years) were enrolled. The participants’ demographic and clinical variables are listed in Table 1.

### 3.2. Developmental Profile

**Figure 1:**
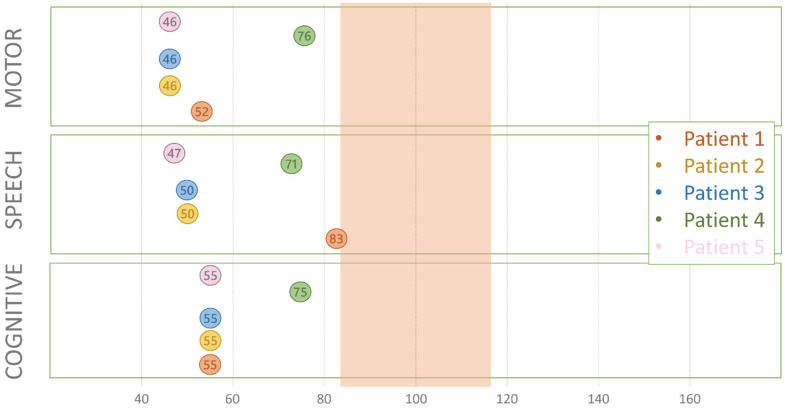
The Bayley Scales of Infant and Toddler Development Third Edition (Bayley-III). The image shows the performance of each patient (identified by a different color) in the three subscales. The standardized mean score is 100 ± 15 standard deviation (highlighted area), values lower than 85 indicate mild impairment, and lower than 70 indicate moderate or severe impairment.

Five children ≤ 42 me raw data supporting the conclusions of this article will be made available by the authors, with the exception of data tracing to single patientsonths old were tested through Bayley-III. Results are shown in Figure 1. Patient n°1 (9 months) had moderate-severe scores in the motor and cognitive area and mild impairment in the language area. Patients n°2, n°3, and n°5 had moderate-severe impairment in all three areas. Patient n°4 had mild impairment in all three areas.

### 3.3. Adaptive Profile

Sixteen individuals > 42 months were evaluated (n°6 to n°21). One (n° 9) had a normal ABC score, 2 had a score between low and moderately low (n°7 and n°12); 13 had very low scores, of whom 11 with IQ 20, the lowest possible score.

For the Communication subscale, only patient n°9 had an adequate adaptive level, one (n°7) had a moderately low level, and 14 had a low adaptive level (of whom 11 scored 20). Median was 20, IQR 13.

For the Daily Living Skills subscale patient n°9 had an adequate adaptive level, 2 moderately low level, and the others 13 had low adaptive level (of whom 9 scored 20). Median was 20, IQR 22.

In the Socialization scale, patient n°9 had an adequate adaptive level, all the others had a low adaptive level (of whom 9 scored 20). Median was 20, IQR 29.5.

**Figure 2:**
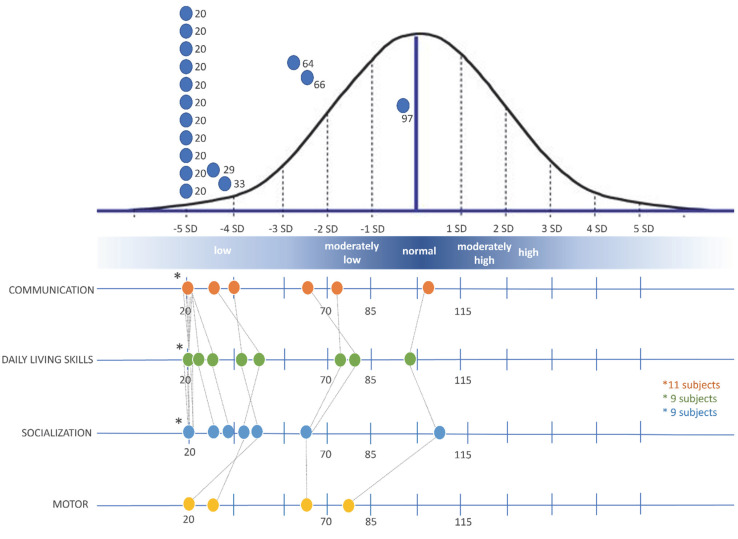
Distribution of Vineland-II ABC and subscales scores.

In the Motor Skills scale, the 4 patients evaluated had low adaptive level (range 20 to 80). Results are pictured in Figure 2.

No statistically significant differences emerged between males and females.

No differences emerged between subjects with or without hearing loss, and those with or without epilepsy.

### 3.4. Behavioral Profile and Repetitive Movements

Twenty-one patients were examined. SSS and BPIs subscales and total scores are reported in Table 2.

No statistically significant differences emerged between males and females.

Subjects < 144 months had higher scores of SIB frequency than those ≥ 144 months (*p* = 0.048).

No differences emerged between subjects with or without hearing loss, and those with or without epilepsy. Individuals with severe low vision had higher AB frequency and severity (*p* = 0.026 and *p* = 0.032) than those without. Moreover, they had higher SSS interference (*p* = 0.027).

For BPIs both SIB-frequency and severity range from 0 to 32; AB frequency ranges 0 to 24, and AB severity 0 to 40.

#### Characterization of Repetitive Behaviors through Direct Video-Analysis

Videos of 19 individuals were collected and analyzed. The most frequently observed stereotypies were head rolling (11/19), hand mouthing (11/19), hand clapping (9/19), grimace (9/19), protrusion of lips (8/19). Less frequently we observed mixed midline hand stereotypies (7/19), oro-buccal stereotypies (7/19), repetitive stimulation of eyes, i.e., eyes pressing or poking (7/19). Other hand stereotypies like hand gaze, flapping, tapping, hand behind the head, and repetitive finger movements were present in 5/19. Comprehensive data are reported in Table S1 of the supplement.

### 3.5. Sensory Profile

**Figure 3:**
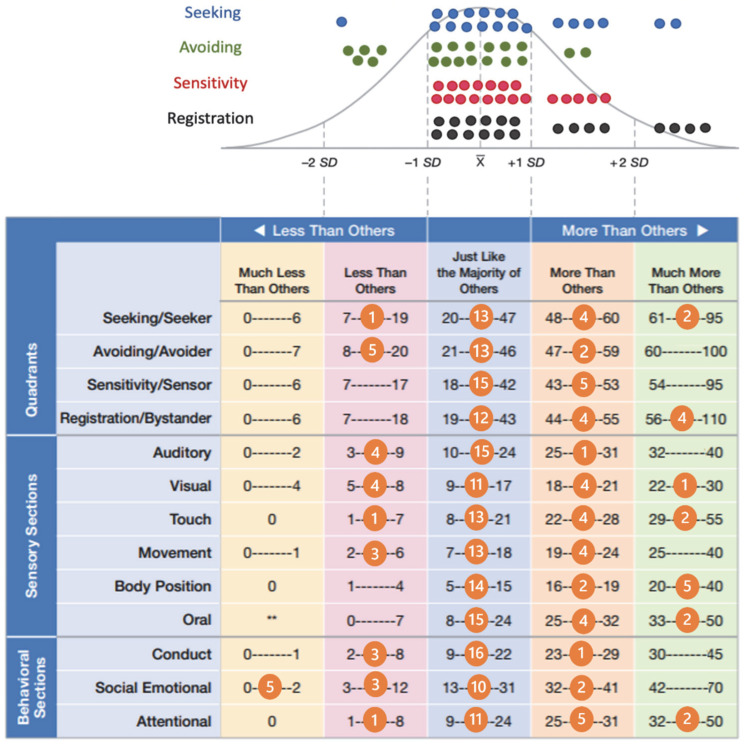
The distribution of the four different sensory processing patterns in the population is shown at the top of the figure. At the bottom of the figure, the number of children falling within the different categories were entered into the C-SP2 scoring sheet.

The C-SP2 was administered to 20 patients. The results are shown in Figure 3.

Half of the population reported values > 1 SD from the mean in at least 1 sensory pattern, more frequently in 2 (6/10) or more (2/10). The other half reported normal values or <1 SD from the mean. Distinctly pathological values (>2 SD) were obtained by patient n°15 and n°16 on the Seeking scale and by patients n°10, n°11, n°16, and n°18 on the Registration scale. Regarding sensory modalities, Body Position showed more clearly pathological scores, resulting in >2 SD in 5 individuals, followed by Touch and Oral in 2 each. Data on individual patient profiles are shown in Table S2 of the supplement.

No statistically significant differences emerged between males and females, nor between < and ≥144 months.

No differences emerged between groups according to the presence of hearing loss, severe hypovision or epilepsy.

### 3.6. Correlations

#### 3.6.1. Adaptive and Behavioral Profile

ABC scores were directly correlated with frequency (ρ = −0.695, *p* = 0.003) and intensity (ρ = −0.679, *p* = 0.004) of stereotypies at SSS. Moreover, with SIB frequency (ρ = −0.704, *p* = 0.002) and severity (ρ = −0.721, *p* = 0.002) at BPIs.

Correlations between the different subscales are reported in Table 3.

#### 3.6.2. Behavioral and Sensory Profile

The sensory Seeking processing pattern positively correlated with number (ρ = 0.518, *p* = 0.019) and intensity (ρ = 0.483, *p* = 0.031) of stereotypies at SSS. Registration pattern positively correlated with stereotypy frequency (ρ = 0.645, *p* = 0.002).

Regarding sensory modalities, Auditory correlated with Global Impairment from stereotypies (ρ = 0.585, *p* = 0.007) and Touch with the number of stereotypies (ρ = 0.488, *p* = 0.029).

Finally, the behavioral section of Attention positively correlated with the frequency of stereotypies (ρ = 0.685, *p* = 0.0008) and the severity of SIB (ρ = 0.481, *p* = 0.032). The complete results are shown in Table S3 of the supplement.

#### 3.6.3. Adaptive and Sensory Profile

Vineland Communication scale negatively correlated with C-SP2 Registration pattern (ρ = −0.522, *p*= 0.038) and with Body Position sensory modality (ρ = −0.546, *p* = 0.028). All data are reported in Table S4 of the supplement.

#### 3.6.4. Correlations with Sleep Disturbances

The Vineland-II Communication scale negatively correlated with the Sleep Breathing Disorders (SBD) subscale (ρ = −0.58, *p* = 0.019), the Disorders of Arousal (DA) subscale (*p* = 0.004), and Total Sleep Disturbance (TSD) (ρ = −0.550, *p* = 0.027) of SDSC.

The DA and SBD scales of the SDSC showed the greatest number of correlations with the SSS and BPIs. DA was positively correlated with SSS frequency (ρ = 0.514, *p* = 0.020), intensity (ρ = 0.589, *p* = 0.006), and interference (ρ = 0.4597, *p* = 0.041); moreover, with SIB frequency (ρ = 0.544, *p* = 0.013) and severity (ρ = 0.605, *p* = 0.005).

The SBD scale was positively correlated with frequency (ρ = 0.608, *p* = 0.004), intensity (ρ = 0.679, *p* = 0.0000), interference (ρ = 0.470, *p* = 0.036), and global impairment (ρ = 0.462, *p* = 0.040); also, with SIB frequency (ρ = 0.612, *p* = 0.004) and severity (ρ = 0.539, *p* = 0.014). The complete results are shown in Table S5 of the supplement.

The Registration scale of C-SP2 was positively correlated with the DA subscale (ρ = 0.512, *p* = 0.021), the Sleep-Wake Transition Disorders (SWTD) subscale (ρ = 0.479, *p* = 0.032), and TSD (ρ = 0.473, *p* = 0.030) of SDSC. DA subscale was also correlated with the sensory disturbances from the Body Position subscale (*p* = 0.048). The complete results are shown in Table S6 of the supplement.

## 4. Discussion

This study carries out a global and contextual analysis of cognitive, adaptive, behavioral, and the sensory profile of PKS on a large sample size.

### 4.1. Developmental and Adaptive Profile

A global psychomotor delay emerged from the study of the younger children, with all except one having moderate/severe impairment in each subscale. The youngest child had borderline scores in the area of language, but this is likely due to the lower skills required for that age. These findings confirm that developmental delay is a common feature in PKS and demonstrate that it occurs from very early childhood. However, it is possible that a selection bias is present because mild phenotypes are more difficult to detect and test at an early age [10,12,34].

In the older group, consistent with literature data [8], all except one had “low” or “moderately low” adaptive level. The Vineland-II sub-scales showed quite homogeneous patterns across abilities in each individual. No clear commonalities emerged among the cohort in this regard. However, the identification of each individual’s strengths and weaknesses, especially those less severe, may be useful to guide rehabilitation therapy.

One child had adequate adaptive level in each assessment area, especially in Socialization and Communication. This child reached the diagnosis because of other features of the syndrome such as typical dysmorphisms and epilepsy. This case confirms the existence of milder neurological phenotypes of PKS [10,12]. Children with subtle phenotypes usually do not come to the attention of the hospital physician unless there are serious complications; this fact can lead to delayed or no diagnosis of the syndrome. On this basis we cannot exclude thus in our study a selection bias related to the severity of the disease.

### 4.2. Behavioral Profile

Stereotypies were found to be frequent and impact on quality of life in almost all patients.

Self-injurious behavior was found in all the subjects, often with a daily frequency; however, the severity was classified by the parents as mild-moderate. Younger children showed a higher frequency, as already found in ASD [35].

Aggressive and destructive behavior occurred rarely and in a mild manner.

Different reasons could explain the presence of stereotypies. Certainly, cognitive disability is one of them as they could be considered to be a primitive form of communication [36,37,38]; unsurprisingly, low scores on the Vineland-II are correlated with greater severity and frequency of stereotypies in our population.

Moreover, both stereotypies and self-injurious behavior could be considered as a form of self-stimulation [39]. However, no significant differences emerged between subjects with and without low vision and hearing loss. A correlation between severe low vision and the presence of hetero-directed aggressive behavior emerged probably as a way of reacting to the external environment, to frustrations, or to the need to express something [38].

Kostanecka’s study already reported the presence of repetitive hand and body movements and self-injurious behavior such as hand or finger biting and head banging [8].

Through the direct video-analysis we defined them clearly: the subjects tended to bring their hands to their mouths, clap their hands, and rock their heads back and forth. Moreover, we observed the presence of oculo-digital phenomena; in one case this phenomenon was so intense to cause a self-enucleation of eyes.

### 4.3. Sensory Profile

The study of the different patterns of sensory response showed in half of the population with values above normal a high neurological threshold and therefore a greater difficulty in capturing the stimuli than the others. This high threshold was followed by a response that was most frequently passive and apathetic (Registration) and, less frequently, active and looking for stimulation (Seeking). The direct correlation of the Seeking scale with the number and intensity of stereotypic behaviors is in line with the known role of stereotypies of alternative sources of sensory stimulation [26,40].

Registration scale inversely correlated with Vineland-II scores; this suggests that this type of disorder might be present in subjects with major neurological impairment. The lower control of the attention mechanisms associated with cognitive impairment could lead to a difficult orientation to sensory stimuli, and the detection of an input [14]. Conversely, a reduced perception of bodily and environmental sensations might be a contributing factor of limited activity participation and socialization [22].

In her population, Kostanecka reported that most subjects refused physical contact, or contact with surrounding objects [8]; the high behavioral responses to tactile stimuli that emerged from our study confirmed and clarified this finding.

Interestingly, no significant correlations with low vision and hearing loss emerged. However, the study of these aspects in children with severe disabilities is often very difficult and the data we collected retrospectively were not homogeneous. Prospective studies in this regard would be necessary to investigate sensory abnormalities from a broader and more comprehensive perspective.

In the hypothesis of “syndrome-related sensory processing profiles” the data obtained do not allow us to define an unequivocal pattern of the syndrome; however, the prevalence of a high threshold brings PKS together with other disorders such as ASD (which can have both hypo- and hypersensitivity) as well as Angelman Syndrome, Down Syndrome, and Cornelia of Lange Syndrome. While subjects with Angelman syndrome have predominantly active responses, those with Cornelia of Lange and Down syndrome have a passive response [22,23]. Moreover, children with PKS often have CNS malformations, including agenesis or dysgenesis of the corpus callosum [7], and this could interfere with the sensory process with a prevalent pattern of Registration (hyporesponsiveness) and a higher score in the Auditory sensory process (both found in our cohort) although the mechanism is not yet clear [24].

The evidence of possible sensory abnormalities in PKS poses the imperative to investigate such children in this sense. Indeed, sensory-based interventions incorporating sensory integration principles might increase their responsiveness to sensation [41]. Moreover, as suggested for Down syndrome, for individuals with disruptive behavior seeking for sensory stimulation, intervention promoting self-regulation could also potentially circumvent maladaptive behavior and promote engagement and participation [21].

### 4.4. Sleep

Sleep disorders had significant correlations with all the profiles studied.

While this could be explained by the fact that subjects with greater neurological impairment also have greater impairment in the sleep domain, the correlation between the communicative sphere and sleep disorders opens the way to speculation, given the known association between these two domains both in individuals with developmental disabilities and neurotypicals [42,43,44].

Moreover, stereotyped behavior and self-harming behavior had strong correlation with sleep disorders. The correlation between these two disorders is known especially with regard to ASD and the link seems to be bidirectional. Indeed, if on the one hand the sleep disorder worsens cognitive skills and stereotypies, on the other hand it has been shown that treatments aimed at reducing stereotypical behaviors also improve sleep quality [45,46]. It is likely that also in subjects with PKS a similar mechanism is present.

In our sample, the sleep disturbances most correlated with sensory alterations are arousal disorders, related to both a hyporesponsiveness pattern and body position modality. These data are not unequivocally interpretable and both hyper- and hyposensitivity are known to be related to sleep in ASD [47].

Further studies are needed to clarify the nature of these correlations and whether sleep alterations may worsen the neurological pattern, or the sleep is disrupted by the presence of sensory abnormalities and stereotypies. Certainly, these data reinforce the recommendation to investigate and treat sleep disorders in these individuals [33].

### 4.5. Limits

One of the main limitations of this study was the impossibility to perform a genotype-phenotype correlation due to the heterogeneity of the genetic data at our disposal. Moreover, as previously mentioned, a selection bias toward more severe phenotypes is possible. Finally, the evolution of individuals over time has not been evaluated.

Further studies will be needed to investigate these aspects.

## 5. Conclusions

The present study is the first attempt to comprehensively and systematically define the developmental, adaptive, behavioral, and sensory profiles of patients with PKS.

Our experience underlines that psychomotor developmental delay and severe adaptive impairment are usually present in PKS, although in our cohort there was a single child with a mild phenotype and normal adaptive profile.

Stereotypies and self-injurious behaviors are frequent and severe in most of the individuals, especially in younger children and in subjects with lower adaptive level. Conversely, hetero-aggressive behaviors are not common.

The analysis of the sensory profile allowed us to individuate pathological modalities of the high threshold type in half of the subjects analyzed and this may have important implications in guiding rehabilitation therapy toward sensory-based interventions that can improve their relationship with the outside world and reduce maladaptive behaviors.

Sleep disorders had significant correlations with all the profiles studied although cause and effect relationships are not completely known. In conclusion, investigating these aspects is essential in guiding rehabilitation therapy through the identification of targeted protocols. Moreover, these data could provide a further step forward in the study of the etiopathogenesis of the syndrome.

## Figures and Tables

**Table 1:** Demographic and clinical characteristics of the population.

	Demographic Data			Clinical Characteristics		
Patient Number	Sex	Genetic Features	Age at the Assessment * (at Bayley -III ^§^)	Epilepsy	Hypoacusis	Severe Hypovision
n° 1	f	47, XX, i(12)(p10) [1]/46, XX, [99] (BK)	0,9 (0,9)	no	no	n/a
n° 2	f	arr [GRCh37] 12p13.33p11.1 (191619_34826574)x2-4 (BAC and SAC)	2,9 (1,6)	no	yes	no
n° 3	f	arr [hg19] 12p13.33p11.1 (163, 679–34,760,977)x2-3 (BAC)	3 (1,5)	no	no	no
n° 4	f	arr12p13.33p11.1 (SAC)	4 (1)	no	yes	yes
n° 5	m	47, XY, i(12p) [7]/46, XY [23] (FK)	4,7 (3,5)	yes	yes	yes
n° 6	f	arr12pterp11.1 (163,593–34,398,316)x2-4 (BAC)	4,10	yes	yes	no
n° 7	m	47,XY, i(12)(p10) [34]/46,XY [16] (FK)	4,9	no	no	no
n° 8	m	arr12pterp11.1 (230,421–34,345,585)×3 (BAC)	5,8	no	no	no
n° 9	f	47,XX, +i(12p)(p10) [22]/46,XX [3] (FK)	6	no	yes	no
n° 10	f	arr [hg19] 12p13.33p11.1 (163, 618–34,756,180)×2–3 (BAC)	8	no	yes	no
n° 11	m	47, XY, +i(12)(p10) [96]/46, XY [4] (FK)	9,10	yes	yes	no
n° 12	m	47, XY, i(12)(p10).ish i(12) (ETV6++)	12	yes	yes	no
n° 13	f	47, XX, i(12)(p10) [2]/46, XX [98] (AK)	13	no	yes	yes
n° 14	f	47, XX, i(12p) (FK)	13	yes	yes	no
n° 15	m	ish CEP 12 (p11.1-q11.1 x2)/CEP 12(p11.1-q11.1 x3) (FF)	13,2	no	n/a	n/a
n° 16	f	47, XX, +i(12)(p10) [14]/46, XX [11] (FK)	13,2	yes	yes	yes
n° 17	m	n/a	14,3	no	n/a	n/a
n° 18	m	47, XY, i(12)(p10)/46, XY (SK)	14,4	no	yes	no
n° 19	f	46, XX [75],47XX+i(12p) [25] (BK)	16	yes	no	yes
n° 20	m	47, XY, i(12)(p10)/46, XY (FK)	16,10	yes	yes	yes
n° 21	m	47,XY, i(12)(p10) (BK)	18,8	yes	yes	no
n° 22	m	47, XY, i(12p) (FK)	28	yes	n/a	no

M = median; m = male; f = female; BK = karyotype on blood; BAC = Array-CGH on blood; SAC = Array-CGH on saliva; FK = karyotype on fibroblasts; AK = karyotype on amniocentesis; SK = karyotype on saliva; FF = FISH on fibroblasts. * Age at the evaluation (years, months). ^§^ Age at the Bayley Scales of Infant and Toddler Development Third Edition administration. The two ages differ because Bayley-III was performed at a different time than the other assessments.

**Table 2:** SSS and BPI subscales and total scores.

Patient n°	SSS						BPIs			
	Number	Frequency	Intensity	Interference	Global Impairment	Total	SIB		AB	
							F	S	F	S
n° 2	3	3	4	1	20	31	3	2	2	2
n° 3	3	3	2	1	0	9	0	0	1	1
n° 4	2	3	1	2	10	18	0	0	0	0
n° 5	3	3	4	3	40	53	13	8	0	0
n° 6	3	3	4	3	30	43	1	1	3	1
n° 7	3	3	3	3	0	12	6	3	10	5
n° 8	3	2	1	0	10	16	4	3	2	2
n° 9	1	1	0	0	0	2	3	2	0	0
n° 10	2	5	4	4	0	15	7	5	3	1
n° 11	3	4	4	2	30	43	7	5	7	3
n° 12	1	1	1	1	10	14	0	0	0	0
n° 13	2	3	4	4	0	13	8	4	0	0
n° 14	2	3	2	2	20	29	5	4	0	0
n° 15	3	4	4	3	40	54	12	6	1	1
n° 16	3	4	4	4	10	25	25	8	1	1
n° 17	2	3	4	4	40	53	11	11	0	0
n° 18	2	4	4	3	20	33	5	3	0	0
n° 19	2	3	4	3	10	22	6	3	0	0
n° 20	2	3	4	4	20	33	10	3	0	0
n° 21	3	4	3	3	30	43	9	5	6	2
n° 22	1	2	2	2	0	7	8	4	9	4
median [IQR]	2 [1]	3 [1]	4 [2]	3 [1]	10 [30]	25 [29]	6 [6]	3 [3]	1 [3]	1 [2]

SSS = severity stereotypy scale; BPIs = behavior problem inventory-short version; SIB = self-injury behavior; AB = aggressive behaviors; F = frequency; S = severity. For SSS: number (0–3 points), frequency (0–5 points), intensity (0–5 points), interference (0–5 points), global impairment (0–50 points), total score (0–68 points).

**Table 3:** Correlations between Vineland subscales and SSS and BPIs subscales.

			Vineland-II		
			Communication	Daily Life	Socialization
SSS	number	ρ	−0.106	−0.119	−0.092
		*p*-value	0.697	0.680	0.736
	frequency	ρ	−0.710 **	−0.494	−0.499 *
		*p*-value	0.002	0.052	0.049
	intensity	ρ	−0.716 **	−0.577 *	−0.605 *
		*p*-value	0.002	0.019	0.013
	interference	ρ	−0.609 *	−0.569 *	−0.584 *
		*p*-value	0.012	0.022	0.018
	global impairment	ρ	−0.428	−0.436	−0.464
		*p*-value	0.098	0.092	0.070
BPIs	SIB frequency	ρ	−0.697 **	−0.705 **	−0.689 **
		*p*-value	0.003	0.002	0.003
	SIB severity	ρ	−0.707 **	−0.624 **	−0.603 *
		*p*-value	0.002	0.010	0.013
	AB frequency	ρ	0.142	0.310	0.325
		*p*-value	0.601	0.242	0.219
	AB severity	ρ	0.164	0.276	0.307
		*p*-value	0.544	0.301	0.248

* *p* < 0.05; ** *p* < 0.01. ρ = rho di Spearman; SSS = stereotypy severity scale; BPIs = behavior problem inventory-short version; SIB = self-injury behaviors; AB = aggressive behaviors.

## Data Availability

The raw data supporting the conclusions of this article will be made available by the authors, with the exception of data tracing to single patients.

## Supplementary Materials

The following are available online at https://www.mdpi.com/article/10.3390/genes13020356/s1. Table S1: Characterization of Repetitive Behaviors through direct video-analysis. Table S2: Sensory Profile-2—Rough results. Table S3: Correlations between Sensory Profile 2 and Behavioral Profile. Table S4: Correlations between Adaptive and Sensory Profile. Table S5: Correlations between Stereotypy Severity Scale, Problem Behavior Inventory—short Version and Sleep Disturbance Scale for Children. Table S6: Correlations between Sensory Profile 2 and Sleep Disturbance Scale for Children.

## References

[1] Larramendy M., Heiskanen M., Wessman M., Ritvanen A., Peltomäki P., Simola K., Kääriäinen H., von Koskull H., Kähkönen M., Knuutila S. (1993). Molecular cytogenetic study of patients with Pallister-Killian syndrome. Hum. Genet..

[2] Karaman B., Kayserili H., Ghanbari A., Uyguner Z.O., Toksoy G., Altunoglu U., Basaran S. (2018). Pallister-Killian syndrome: Clinical, cytogenetic and molecular findings in 15 cases. Mol. Cytogenet..

[3] Blyth M., Maloney V., Beal S., Collinson M., Huang S., Crolla J., Temple I.K., Baralle D. (2015). Pallister-Killian syndrome: A study of 22 British patients. J. Med. Genet..

[4] Conlin L.K., Kaur M., Izumi K., Campbell L., Wilkens A., Clark D., Deardorff M.A., Zackai E.H., Pallister P., Hakonarson H. (2012). Utility of SNP arrays in detecting, quantifying, and determining meiotic origin of tetrasomy 12p in blood from individuals with Pallister-Killian syndrome. Am. J. Med. Genet. Part A.

[5] Ricci E., Bonfatti R., Rocca A., Sperti G., Cagnazzo V., Vignoli A., Cocchi G., Cordelli D.M. (2019). Myoclonic epilepsy with photosensitivity in infants with Pallister-Killian Syndrome. Eur. J. Paediatr. Neurol..

[6] Izumi K., Krantz I.D. (2014). Pallister-Killian syndrome. Am. J. Med. Genet. Part C Semin. Med. Genet..

[7] Poulton C., Baynam G., Yates C., Alinejad-Rokny H., Williams S., Wright H., Woodward K.J., Sivamoorthy S., Peverall J., Shipman P. (2018). A review of structural brain abnormalities in Pallister-Killian syndrome. Mol. Genet. Genom. Med..

[8] Kostanecka A., Close L.B., Izumi K., Krantz I.D., Pipan M. (2012). Developmental and behavioral characteristics of individuals with Pallister-Killian syndrome. Am. J. Med. Genet. Part A.

[9] Stalker H.J., Gray B.A., Bent-Williams A., Zori R.T. (2006). High cognitive functioning and behavioral phenotype in Pallister-Killian syndrome. Am. J. Med. Genet. Part A.

[10] Genevieve D., Cormier-Daire V., Sanlaville D., Faivre L., Gosset P., Allart L., Picq M., Munnich A., Romana S., de Blois M. (2003). Mild phenotype in a 15-year-old boy with Pallister-Killian syndrome. Am. J. Med. Genet..

[11] Schaefer G.B., Jochar A., Muneer R., Sanger W.G. (2008). Clinical variability of tetrasomy 12p. Clin. Genet..

[12] Bielanska M.M., Khalifa M.M., Duncan A.M.V. (1996). Pallister-Killian syndrome: A mild case diagnosed by fluorescence in situ hybridization. Review of the literature and expansion of the phenotype. Am. J. Med. Genet..

[13] Miller L.J., Anzalone M.E., Lane S.J., Cermak S.A., Osten E.T. (2007). Concept Evolution in Sensory Integration: A Proposed Nosology for Diagnosis. Am. J. Occup. Ther..

[14] Little L.M., Dean E., Tomchek S.D., Dunn W. (2017). Classifying sensory profiles of children in the general population. Child. Care. Health Dev..

[15] Fetta A., Carati E., Moneti L., Pignataro V., Angotti M., Bardasi M.C., Cordelli D.M., Franzoni E., Parmeggiani A. (2021). Relationship between Sensory Alterations and Repetitive Behaviours in Children with Autism Spectrum Disorders: A Parents’ Questionnaire Based Study. Brain Sci..

[16] Glod M., Riby D.M., Rodgers J. (2020). Sensory processing profiles and autistic symptoms as predictive factors in autism spectrum disorder and Williams syndrome. J. Intellect. Disabil. Res..

[17] Simpson K., Adams D., Alston-Knox C., Heussler H.S., Keen D. (2019). Exploring the Sensory Profiles of Children on the Autism Spectrum Using the Short Sensory Profile-2 (SSP-2). J. Autism Dev. Disord..

[18] Thye M.D., Bednarz H.M., Herringshaw A.J., Sartin E.B., Kana R.K. (2018). The impact of atypical sensory processing on social impairments in autism spectrum disorder. Dev. Cogn. Neurosci..

[19] Little L.M., Dean E., Tomchek S., Dunn W. (2018). Sensory Processing Patterns in Autism, Attention Deficit Hyperactivity Disorder, and Typical Development. Phys. Occup. Ther. Pediatr..

[20] Bataglia A. (2011). Sensory impairment in mental retardation: A potential role for NGF. Arch. Ital. Biol..

[21] Will E.A., Daunhauer L.A., Fidler D.J., Raitano Lee N., Rosenberg C.R., Hepburn S.L. (2019). Sensory Processing and Maladaptive Behavior: Profiles Within the Down Syndrome Phenotype. Phys. Occup. Ther. Pediatr..

[22] Wuang Y.P., Su C.Y. (2011). Correlations of sensory processing and visual organization ability with participation in school-aged children with Down syndrome. Res. Dev. Disabil..

[23] Heald M., Adams D., Oliver C. (2020). Profiles of atypical sensory processing in Angelman, Cornelia de Lange and Fragile X syndromes. J. Intellect. Disabil. Res..

[24] Demopoulos C., Arroyo M.S., Dunn W., Strominger Z., Sherr E.H., Marco E. (2015). Individuals with agenesis of the corpus callosum show sensory processing differences as measured by the sensory profile. Neuropsychology.

[25] Royston R., Oliver C., Moss J., Adams D., Berg K., Burbidge C., Howlin P., Nelson L., Stinton C., Waite J. (2018). Brief Report: Repetitive Behaviour Profiles in Williams syndrome: Cross Syndrome Comparisons with Prader–Willi and Down syndromes. J. Autism Dev. Disord..

[26] Schulz S.E., Stevenson R.A. (2019). Sensory hypersensitivity predicts repetitive behaviours in autistic and typically-developing children. Autism.

[27] Miller J.M., Singer H.S., Bridges D.D., Waranch H.R. (2006). Behavioral therapy for treatment of stereotypic movements in nonautistic children. J. Child Neurol..

[28] Rojahn J., Rowe E.W., Sharber A.C., Hastings R., Matson J.L., Didden R., Kroes D.B.H., Dumont E.L.M. (2012). The Behavior Problems Inventory-Short Form for individuals with intellectual disabilities: Part I: Development and provisional clinical reference data. J. Intellect. Disabil. Res..

[29] Rojahn J., Rowe E.W., Sharber A.C., Hastings R., Matson J.L., Didden R., Kroes D.B.H., Dumont E.L.M. (2012). The Behavior Problems Inventory-Short Form for individuals with intellectual disabilities: Part II: Reliability and validity. J. Intellect. Disabil. Res..

[30] Dunn W. (2014). Sensory Profile 2.

[31] Fernandez-Alvarez E., Arzimanoglou A., Tolosa E. (2005). Paediatric Movement Disorders: Progress in Understanding.

[32] Temudo T., Oliveira P., Santos M., Dias K., Vieira J., Moreira A., Calado E., Carrilho I., Oliveira G., Levy A. (2007). Stereotypies in Rett syndrome: Analysis of 83 patients with and without detected MECP2 mutations. Neurology.

[33] Fetta A., Di Pisa V., Ruscelli M., Soliani L., Sperti G., Ubertiello S., Ricci E., Mainieri G., Rocca A., Mancardi M.M. (2021). Sleep in Children With Pallister Killian Syndrome: A Prospective Clinical and Videopolysomnographic Study. Front. Neurol..

[34] Wilkens A., Liu H., Park K., Campbell L.B., Jackson M., Kostanecka A., Pipan M., Izumi K., Pallister P., Krantz I.D. (2012). Novel clinical manifestations in Pallister-Killian syndrome: Comprehensive evaluation of 59 affected individuals and review of previously reported cases. Am. J. Med. Genet. Part A.

[35] Melo C., Ruano L., Jorge J., Pinto Ribeiro T., Oliveira G., Azevedo L., Temudo T. (2020). Prevalence and determinants of motor stereotypies in autism spectrum disorder: A systematic review and meta-analysis. Autism.

[36] Bartak L., Rutter M. (1976). Differences between mentally retarded and normally intelligent autistic children. J. Autism Child. Schizophr..

[37] Lanzarini E., Pruccoli J., Grimandi I., Spadoni C., Angotti M., Pignataro V., Sacrato L., Franzoni E., Parmeggiani A. (2021). Phonic and Motor Stereotypies in Autism Spectrum Disorder: Video Analysis and Neurological Characterization. Brain Sci..

[38] Gal E., Dyck M.J., Passmore A. (2009). The relationship between stereotyped movements and self-injurious behavior in children with developmental or sensory disabilities. Res. Dev. Disabil..

[39] Symons F.J., Sperry L.A., Dropik P.L., Bodfish J.W. (2005). The early development of stereotypy and self-injury: A review of research methods. J. Intellect. Disabil. Res..

[40] Lovaas I., Newsom C., Hickman C. (1987). Self-stimulatory behavior and perceptual reinforcement. J. Appl. Behav. Anal..

[41] Zimmer M., Desch L. (2012). Sensory integration therapies for children with developmental and behavioral disorders. Pediatrics.

[42] Elia M., Ferri R., Musumeci S.A., Del Gracco S., Bottitta M., Scuderi C., Miano G., Panerai S., Bertrand T., Grubar J.C. (2000). Sleep in subjects with autistic disorder: A neurophysiological and psychological study. Brain Dev..

[43] Taylor M.A., Schreck K.A., Mulick J.A. (2012). Sleep disruption as a correlate to cognitive and adaptive behavior problems in autism spectrum disorders. Res. Dev. Disabil..

[44] Schreck K.A., Mulick J.A., Smith A.F. (2004). Sleep problems as possible predictors of intensified symptoms of autism. Res. Dev. Disabil..

[45] Hunter J.E., McLay L.K., France K.G., Blampied N.M. (2021). Sleep and stereotypy in children with autism: Effectiveness of function-based behavioral treatment. Sleep Med..

[46] Backer N.B.A., Alzawad M., Habibullah H., Bashir S. (2018). The relationship between sleep and cognitive performance in autism spectrum disorder (ASD): A pilot study. Children.

[47] Tzischinsky O., Meiri G., Manelis L., Bar-Sinai A., Flusser H., Michaelovski A., Zivan O., Ilan M., Faroy M., Menashe I. (2018). Sleep disturbances are associated with specific sensory sensitivities in children with autism. Mol. Autism.

