# Supplementary material for: Cognitive, Behavioral, and Sensory Profile of Pallister–Killian Syndrome: A Prospective Study of 22 Individuals
Source: Genes (Basel). 2022 Feb 16;13(2):356. doi: 10.3390/genes13020356 (PMC8872298; doi:10.3390/genes13020356)
Supplement: Supplementary file 1 [file genes-13-00356-s001.zip › genes-1557687-SI.pdf]

**Table S1. Characterization of Repetitive Behaviors through Direct Video Analysis**

| Patient number | RH | R | G | B | PL | RCE | ER | JHW | JHC | M | SHM | HP | P | HBN | C | TF | F | S | HG | T | MHM | RFM | CAM | HBH | HT | OBS | EPP |
|---|---|---|---|---|---|---|---|---|---|---|---|---|---|---|---|---|---|---|---|---|---|---|---|---|---|---|---|
| **n° 1** | 0 | 0 | 1 | 0 | 1 | 0 | 0 | 1 | 0 | 0 | 1 | 0 | 0 | 0 | 1 | 0 | 1 | 1 | 1 | 0 | 0 | 1 | 1 | 0 | 0 | 1 | 0 |
| **n°2** | 0 | 0 | 0 | 0 | 0 | 0 | 0 | 0 | 1 | 0 | 0 | 0 | 0 | 0 | 0 | 0 | 0 | 0 | 0 | 0 | 1 | 0 | 0 | 0 | 0 | 1 | 0 |
| **n°3** | 1 | 0 | 0 | 0 | 1 | 0 | 0 | 1 | 0 | 0 | 1 | 0 | 1 | 0 | 0 | 0 | 0 | 0 | 1 | 0 | 0 | 1 | 0 | 1 | 0 | 1 | 1 |
| **n°5** | 1 | 0 | 0 | 0 | 0 | 0 | 0 | 0 | 0 | 0 | 1 | 0 | 0 | 0 | 0 | 0 | 0 | 0 | 0 | 0 | 0 | 0 | 1 | 0 | 0 | 0 | 0 |
| **n°6** | 1 | 0 | 1 | 0 | 0 | 0 | 0 | 0 | 0 | 1 | 1 | 0 | 0 | 0 | 1 | 0 | 0 | 1 | 1 | 1 | 1 | 1 | 0 | 1 | 0 | 1 | 1 |
| **n°7** | 0 | 0 | 0 | 0 | 0 | 0 | 0 | 0 | 1 | 1 | 1 | 0 | 0 | 0 | 1 | 0 | 0 | 0 | 0 | 0 | 0 | 0 | 0 | 0 | 0 | 0 | 0 |
| **n°8** | 1 | 1 | 0 | 0 | 1 | 0 | 0 | 1 | 0 | 0 | 0 | 0 | 0 | 0 | 0 | 0 | 0 | 0 | 0 | 1 | 0 | 0 | 0 | 0 | 0 | 0 | 0 |
| **n°9** | 1 | 1 | 0 | 0 | 1 | 0 | 1 | 1 | 1 | 1 | 1 | 0 | 0 | 0 | 0 | 0 | 0 | 0 | 0 | 0 | 1 | 0 | 1 | 1 | 0 | 0 | 0 |
| **n°10** | 1 | 0 | 1 | 0 | 0 | 0 | 0 | 0 | 1 | 1 | 1 | 0 | 0 | 0 | 0 | 0 | 1 | 0 | 1 | 0 | 1 | 0 | 0 | 0 | 0 | 1 | 0 |
| **n°11** | 0 | 0 | 1 | 0 | 1 | 0 | 0 | 0 | 1 | 1 | 1 | 0 | 0 | 0 | 0 | 0 | 1 | 0 | 0 | 0 | 1 | 1 | 0 | 0 | 0 | 0 | 1 |
| **n°14** | 1 | 0 | 0 | 0 | 0 | 0 | 0 | 0 | 0 | 0 | 0 | 0 | 0 | 0 | 0 | 0 | 0 | 0 | 0 | 1 | 0 | 0 | 0 | 0 | 0 | 1 | 0 |
| **n°15** | 0 | 0 | 0 | 0 | 0 | 0 | 0 | 0 | 0 | 1 | 0 | 0 | 0 | 0 | 0 | 0 | 0 | 0 | 0 | 0 | 0 | 0 | 1 | 0 | 0 | 0 | 1 |
| **n°16** | 0 | 0 | 0 | 0 | 1 | 0 | 0 | 1 | 0 | 1 | 0 | 0 | 0 | 0 | 0 | 0 | 0 | 0 | 0 | 0 | 0 | 0 | 0 | 1 | 0 | 0 | 0 |
| **n°17** | 0 | 0 | 0 | 0 | 0 | 0 | 0 | 0 | 0 | 1 | 0 | 0 | 0 | 0 | 1 | 0 | 0 | 1 | 0 | 0 | 1 | 0 | 0 | 0 | 0 | 0 | 0 |
| **n°18** | 1 | 0 | 0 | 0 | 0 | 0 | 0 | 0 | 1 | 0 | 0 | 0 | 0 | 0 | 0 | 0 | 0 | 0 | 0 | 1 | 0 | 0 | 0 | 0 | 0 | 0 | 0 |
| **n°19** | 0 | 0 | 0 | 0 | 0 | 0 | 0 | 0 | 0 | 0 | 0 | 0 | 0 | 0 | 0 | 0 | 1 | 1 | 1 | 0 | 0 | 1 | 1 | 0 | 0 | 0 | 0 |
| **n°20** | 1 | 0 | 1 | 0 | 0 | 0 | 0 | 0 | 0 | 1 | 0 | 0 | 0 | 0 | 0 | 0 | 1 | 0 | 0 | 0 | 0 | 0 | 0 | 0 | 0 | 1 | 1 |
| **n°21** | 0 | 0 | 1 | 0 | 0 | 0 | 0 | 0 | 0 | 0 | 0 | 0 | 0 | 0 | 0 | 0 | 0 | 0 | 0 | 0 | 0 | 0 | 0 | 0 | 0 | 0 | 0 |
| **n°22** | 1 | 1 | 1 | 0 | 0 | 0 | 0 | 0 | 0 | 1 | 1 | 0 | 0 | 0 | 0 | 0 | 0 | 0 | 0 | 0 | 1 | 1 | 0 | 1 | 0 | 0 | 1 |
| total | 10 | 3 | 7 | 0 | 6 | 0 | 1 | 5 | 6 | 10 | 9 | 0 | 1 | 0 | 4 | 0 | 5 | 4 | 5 | 4 | 7 | 6 | 5 | 5 | 0 | 7 | 6 |

RH: rolling head; R:retropulsion; G:grimace; B:bruxism; PL: protrusion of lips; RCE: repetitive closure of eyes; ER: eye rolling; JHW: joined hands washing; JHC: joined hands clapping; M: mouthing; SHM: separated hands mouthing; HP: hair pulling; PR: pill rolling; HBN: one hand behind the neck; C: castanets; TF: twisting two or three fingers; F: flapping; S: sevillana; HG: hand gaze; T: tapping; MHM: mixed midline hand movements; RFM: repetitive finger movements; CAM: complex arm movement; HBH: hand behind head; HT: hair twirling; OBS: oro-buccal stereotypies; EPP: eyes pressing or poking.

**Table S2. Sensory Profile-2 Rough results**

| patient | seeking | avoiding | sensitivity | registration | auditory | visual | touch | movement | body position | oral | conduct | social emotion | attentional |
|---|---|---|---|---|---|---|---|---|---|---|---|---|---|
| **n° 2** | 26 | 30 | 40 | 41 | 14 | 17 | 11 | 7 | 13 | 18 | 12 | 26 | 15 |
| **n° 3** | **24** | 33 | 50 | 51 | 13 | 16 | 22 | 2 | 26 | 27 | 11 | 25 | 24 |
| **n° 4** | **49** | 35 | 29 | 36 | 15 | 19 | 13 | 16 | 15 | 19 | 20 | 21 | 26 |
| **n° 5** | **27** | 23 | 41 | 36 | 16 | 13 | 17 | 6 | 10 | 21 | 15 | 16 | 17 |
| **n° 6** | **48** | 24 | 43 | 30 | 18 | 23 | 26 | 20 | 9 | 37 | 11 | 5 | 7 |
| **n° 7** | **41** | 41 | 40 | 33 | 17 | 17 | 15 | 16 | 9 | 15 | 17 | 24 | 25 |
| **n° 8** | **37** | 23 | 26 | 29 | 12 | 11 | 18 | 9 | 10 | 21 | 9 | 10 | 15 |
| **n° 9** | **25** | 24 | 22 | 31 | 8 | 16 | 11 | 10 | 11 | 15 | 15 | 18 | 10 |
| **n° 10** | **28** | 19 | 32 | 62 | 7 | 5 | 12 | 16 | 17 | 21 | 20 | 17 | 33 |
| **n° 11** | **56** | 38 | 43 | 58 | 30 | 19 | 29 | 16 | 27 | 40 | 15 | 0 | 27 |
| **n° 12** | **25** | 24 | 23 | 22 | 10 | 6 | 10 | 9 | 8 | 12 | 13 | 16 | 14 |
| **n° 13** | **25** | 15 | 30 | 32 | 9 | 6 | 6 | 7 | 21 | 22 | 10 | 0 | 21 |
| **n° 14** | **53** | 23 | 40 | 48 | 22 | 11 | 21 | 23 | 14 | 21 | 19 | 9 | 28 |
| **n° 15** | **61** | 42 | 42 | 45 | 19 | 11 | 28 | 31 | 13 | 19 | 15 | 25 | 35 |
| **n° 16** | **62** | 51 | 56 | 57 | 19 | 21 | 40 | 14 | 17 | 29 | 26 | 33 | 41 |
| **n° 17** | **23** | 32 | 51 | 45 | 18 | 14 | 11 | 11 | 21 | 26 | 10 | 25 | 19 |
| **n° 18** | **30** | 53 | 37 | 57 | 14 | 21 | 26 | 19 | 24 | 11 | 14 | 33 | 27 |
| **n° 19** | **20** | 13 | 23 | 34 | 7 | 14 | 10 | 11 | 15 | 9 | 7 | 0 | 11 |
| **n° 20** | **26** | 8 | 29 | 29 | 15 | 8 | 17 | 10 | 9 | 29 | 2 | 0 | 10 |
| **n° 21** | **16** | 12 | 22 | 24 | 10 | 9 | 9 | 4 | 6 | 14 | 6 | 1 | 16 |

**Table S3. Correlations between Sensory Profile 2 and behavioral profile**

| Variable | | | SSS | | | | | BPIs | | | |
|---|---|---|---|---|---|---|---|---|---|---|---|
| | | | number | frequency | intensity | interference | global impairmant | SIB frequency | SIB severity | AB frequency | AB severity |
| Sensory Profile 2 | seeking | ρ | 0.3386 | 0.3297 | 0.1066 | 0.005450 | 0.1556 | 0.1099 | 0.1643 | 0.2703 | 0.2802 |
| | | p-value | 0.1443 | 0.1558 | 0.6547 | 0.9818 | 0.5124 | 0.6445 | 0.4889 | 0.2490 | 0.2315 |
| | avoiding | ρ | 0.2645 | 0.2467 | 0.02561 | -0.1395 | 0.07249 | -0.08321 | -0.006868 | 0.1697 | 0.2426 |
| | | p-value | 0.2598 | 0.2944 | 0.9147 | 0.5574 | 0.7614 | 0.7273 | 0.9771 | 0.4746 | 0.3027 |
| | sensitivity | ρ | 0.5180 * | 0.3675 | 0.4827 * | 0.2787 | 0.3374 | 0.2908 | 0.3712 | 0.2319 | 0.2414 |
| | | p-value | 0.01930 | 0.1109 | 0.03109 | 0.2342 | 0.1457 | 0.2135 | 0.1071 | 0.3253 | 0.3052 |
| | registration | ρ | 0.1471 | 0.6449 ** | 0.4049 | 0.2020 | 0.04081 | 0.2141 | 0.3810 | 0.1089 | 0.1037 |
| | | p-value | 0.5361 | 0.002139 | 0.07659 | 0.3932 | 0.8644 | 0.3647 | 0.09747 | 0.6477 | 0.6636 |
| | auditory | ρ | 0.4432 | 0.2594 | 0.2457 | 0.1145 | 0.5845 ** | 0.2971 | 0.3530 | 0.1724 | 0.2050 |
| | | p-value | 0.05031 | 0.2694 | 0.2965 | 0.6308 | 0.006802 | 0.2034 | 0.1268 | 0.4675 | 0.3859 |
| | visual | ρ | 0.3442 | 0.1093 | 0.1029 | -0.1665 | 0.1258 | -0.2226 | -0.1970 | 0.1892 | 0.2123 |
| | | p-value | 0.1372 | 0.6466 | 0.6658 | 0.4828 | 0.5972 | 0.3456 | 0.4051 | 0.4244 | 0.3689 |
| | touch | ρ | 0.4877 * | 0.3795 | 0.2061 | -0.01792 | 0.2798 | 0.1270 | 0.1583 | 0.2608 | 0.2836 |
| | | p-value | 0.02915 | 0.09889 | 0.3834 | 0.9402 | 0.2321 | 0.5936 | 0.5051 | 0.2667 | 0.2255 |
| | movement | ρ | -0.08556 | 0.3625 | 0.1942 | 0.1898 | 0.1932 | 0.03563 | 0.1251 | 0.08421 | -0.005304 |
| | | p-value | 0.7199 | 0.1162 | 0.4119 | 0.4227 | 0.4144 | 0.8814 | 0.5994 | 0.7241 | 0.9823 |
| | body position | ρ | -0.06614 | 0.3910 | 0.3168 | 0.1517 | -0.1331 | 0.06924 | 0.2069 | -0.1333 | -0.1063 |
| | | p-value | 0.7817 | 0.08824 | 0.1735 | 0.5233 | 0.5760 | 0.7718 | 0.3815 | 0.5752 | 0.6556 |
| | oral | ρ | 0.3387 | 0.1582 | 0.3203 | 0.2835 | 0.1738 | 0.2493 | 0.2515 | 0.2019 | 0.1668 |
| | | p-value | 0.1440 | 0.5052 | 0.1686 | 0.2258 | 0.4636 | 0.2891 | 0.2847 | 0.3932 | 0.4821 |
| | conduct | ρ | 0.001677 | 0.2810 | -0.1069 | -0.03553 | -0.1816 | 0.003410 | 0.1319 | 0.09510 | 0.06404 |
| | | p-value | 0.9944 | 0.2301 | 0.6537 | 0.8818 | 0.4436 | 0.9886 | 0.5794 | 0.6900 | 0.7885 |
| | social emotion | ρ | 0.1393 | 0.1618 | -0.02183 | -0.07271 | -0.04100 | -0.05349 | 0.006506 | 0.008103 | 0.09310 |
| | | p-value | 0.5579 | 0.4957 | 0.9272 | 0.7606 | 0.8637 | 0.8228 | 0.9783 | 0.9730 | 0.6962 |
| | attentional | ρ | 0.1955 | 0.6850 *** | 0.1789 | 0.2630 | -0.01039 | 0.3420 | 0.4814 * | 0.1697 | 0.1857 |
| | | p-value | 0.4089 | 8.597e -4 | 0.4504 | 0.2627 | 0.9653 | 0.1399 | 0.03164 | 0.4743 | 0.4331 |

* $p < 0.05$. ** $p < 0.01$. ***$p < 0.001$.

**Table S4. Correlations between Adaptive and Sensory Profile**

| Variable | | | Sensory Profile 2 | | | | | | | | | | | | |
|---|---|---|---|---|---|---|---|---|---|---|---|---|---|---|---|
| | | | seeking | avoiding | sensitivity | registration | auditory | visual | touch | movement | body position | oral | conduct | social emotion | attentional |
| Vineland-II | Communication | ρ | -0.03228 | 0.1382 | -0.3102 | -0.5215 * | -0.2080 | 0.1546 | -0.1434 | -0.1779 | -0.5460 * | -0.2101 | 0.07537 | 0.1515 | -0.4906 |
| | | p-value | 0.9055 | 0.6098 | 0.2422 | 0.03831 | 0.4395 | 0.5676 | 0.5963 | 0.5097 | 0.02868 | 0.4347 | 0.7814 | 0.5753 | 0.05366 |
| | Daily Lining | ρ | 0.03901 | 0.1220 | -0.2324 | -0.3005 | -0.2210 | 0.08470 | -0.06984 | -0.07819 | -0.3906 | -0.06185 | 0.2310 | 0.06376 | -0.3798 |
| | | p-value | 0.8860 | 0.6527 | 0.3864 | 0.2582 | 0.4107 | 0.7551 | 0.7972 | 0.7735 | 0.1346 | 0.8200 | 0.3894 | 0.8145 | 0.1468 |
| | Sociality | ρ | 0.04230 | 0.1205 | -0.2489 | -0.2942 | -0.2286 | 0.07092 | -0.07071 | -0.1003 | -0.3812 | -0.07739 | 0.2295 | 0.07528 | -0.3557 |
| | | p-value | 0.8764 | 0.6567 | 0.3526 | 0.2687 | 0.3945 | 0.7941 | 0.7947 | 0.7118 | 0.1451 | 0.7758 | 0.3924 | 0.7817 | 0.1763 |

* $p < 0.05$.

**Table S5. Correlations between Stereotipy Severity Scale, Problem Behavior Inventory – short version and Sleep Disturbance Scale for Children**

| Variable | | | Stereotypy Severity Scale | | | | | | Problem Behavior Inventory – short version | | | |
|---|---|---|---|---|---|---|---|---|---|---|---|---|
| | | | number | frequency | intensity | interference | global | SSS | SIB | SIB | AB | AB |
| Sleep Disturbance Scale for Children | DIMS | ρ | 0.077 | 0.330 | 0.401 | 0.321 | 0.433 | 0.182 | 0.649** | 0.537* | -0.085 | -0.043 |
| | | p-value | 0.747 | 0.155 | 0.080 | 0.168 | 0.056 | 0.443 | 0.002 | 0.015 | 0.723 | 0.858 |
| | SBD | ρ | 0.311 | 0.608** | 0.679*** | 0.470* | 0.462* | 0.109 | 0.612** | 0.540* | 0.276 | 0.234 |
| | | p-value | 0.182 | 0.004 | 0.001 | 0.036 | 0.040 | 0.647 | 0.004 | 0.014 | 0.239 | 0.320 |
| | DA | ρ | -0.130 | 0.514* | 0.589** | 0.460* | 0.365 | 0.409 | 0.544* | 0.605** | -0.134 | -0.193 |
| | | p-value | 0.586 | 0.020 | 0.006 | 0.041 | 0.114 | 0.074 | 0.013 | 0.005 | 0.573 | 0.416 |
| | SWTD | ρ | 0.066 | 0.356 | 0.194 | 0.071 | 0.342 | 0.287 | 0.190 | 0.365 | -0.041 | -0.097 |
| | | p-value | 0.782 | 0.123 | 0.413 | 0.766 | 0.140 | 0.221 | 0.423 | 0.113 | 0.865 | 0.683 |
| | DOES | ρ | -0.102 | 0.034 | 0.326 | 0.226 | 0.195 | 0.414 | 0.229 | 0.275 | -0.174 | -0.194 |
| | | p-value | 0.669 | 0.888 | 0.161 | 0.338 | 0.410 | 0.069 | 0.332 | 0.241 | 0.464 | 0.413 |
| | SHY | ρ | -0.163 | -0.262 | -0.123 | -0.143 | -0.094 | 0.082 | -0.169 | -0.243 | -0.112 | -0.163 |
| | | p-value | 0.493 | 0.264 | 0.607 | 0.548 | 0.694 | 0.731 | 0.475 | 0.301 | 0.637 | 0.491 |
| | TOTAL | ρ | 0.001 | 0.368 | 0.400 | 0.215 | 0.354 | 0.414 | 0.467* | 0.467* | -0.089 | -0.084 |
| | | p-value | 0.997 | 0.111 | 0.081 | 0.362 | 0.126 | 0.070 | 0.038 | 0.038 | 0.709 | 0.726 |

* $p < 0.05$. ** $p < 0.01$. ***$p < 0.001$.

**Table S6. Correlations between Sensory Profile 2 and Sleep Disturbance Scale for Children**

| Variable | | | Sensory Profile - 2 | | | | | | | | | | | | |
|---|---|---|---|---|---|---|---|---|---|---|---|---|---|---|---|
| | | | **seeking** | **avoiding** | **sensitivity** | **registration** | **auditory** | **visual** | **touch** | **movement** | **body** | **oral** | **conduct** | **social** | **attentional** |
| **Sleep Disturbance Scale for Children** | **DIMS** | ϱ | -0.130 | 0.044 | 0.156 | 0.202 | 0.172 | -0.081 | 0.043 | -0.113 | 0.034 | 0.047 | -0.051 | 0.216 | 0.084 |
| | | p-value | 0.586 | 0.854 | 0.511 | 0.393 | 0.468 | 0.734 | 0.858 | 0.636 | 0.885 | 0.844 | 0.831 | 0.360 | 0.724 |
| | **SBD** | ϱ | -0.005 | -0.054 | 0.293 | 0.329 | 0.179 | -0.058 | 0.169 | -0.083 | 0.050 | 0.230 | -0.025 | 0.006 | 0.143 |
| | | p-value | 0.983 | 0.820 | 0.209 | 0.157 | 0.451 | 0.807 | 0.475 | 0.728 | 0.836 | 0.330 | 0.917 | 0.979 | 0.548 |
| | **DA** | ϱ | 0.197 | -0.056 | 0.239 | 0.512 * | 0.242 | -0.304 | 0.208 | 0.376 | 0.447* | 0.244 | 0.098 | -0.223 | 0.429 |
| | | p-value | 0.405 | 0.813 | 0.310 | 0.021 | 0.305 | 0.193 | 0.378 | 0.103 | 0.048 | 0.299 | 0.681 | 0.344 | 0.059 |
| | **SWTD** | ϱ | 0.074 | 0.048 | 0.201 | 0.480* | 0.073 | -0.068 | 0.194 | 0.270 | 0.372 | 0.018 | 0.163 | 0.028 | 0.336 |
| | | p-value | 0.758 | 0.840 | 0.395 | 0.032 | 0.759 | 0.776 | 0.413 | 0.250 | 0.106 | 0.939 | 0.492 | 0.905 | 0.148 |
| | **DOES** | ϱ | -0.064 | -0.169 | 0.376 | 0.349 | 0.280 | -0.194 | 0.058 | -0.055 | 0.199 | 0.331 | 0.070 | -0.120 | 0.094 |
| | | p-value | 0.788 | 0.476 | 0.102 | 0.132 | 0.232 | 0.412 | 0.807 | 0.819 | 0.401 | 0.155 | 0.769 | 0.614 | 0.695 |
| | **SHY** | ϱ | 0.018 | -0.299 | -0.283 | -0.183 | -0.247 | -0.029 | - | -0.110 | -0.285 | 0.086 | 0.116 | -0.055 | -0.380 |
| | | p-value | 0.939 | 0.201 | 0.226 | 0.439 | 0.294 | 0.903 | 0.668 | 0.644 | 0.223 | 0.719 | 0.627 | 0.818 | 0.098 |
| | **TOTAL** | ϱ | 0.028 | -0.128 | 0.194 | 0.473* | 0.164 | -0.242 | 0.156 | 0.074 | 0.237 | 0.185 | 0.101 | 0.017 | 0.230 |
| | | p-value | 0.908 | 0.590 | 0.413 | 0.035 | 0.489 | 0.305 | 0.511 | 0.757 | 0.314 | 0.435 | 0.673 | 0.944 | 0.330 |

* $p < 0.05$.
